# Development of Silafluofen-Based Termiticides in Japan and Thailand

**DOI:** 10.3390/insects2040532

**Published:** 2011-12-08

**Authors:** Yoshio Katsuda, Yoshihiro Minamite, Charunee Vongkaluang

**Affiliations:** 1 Dainihon Jochugiku Co., Ltd., 1-11, 1-Chome, Daikoku-cho, Toyonaka, Osaka 561-0827, Japan;E-Mail: y.katsuda@kincho.co.jp; 2 Royal Forest Department, Bangkok 10900, Thailand; E-Mail: chaisavong@gmail.com

**Keywords:** pyrethroid, silafluofen, termiticide, fish toxicity

## Abstract

With the advancement from natural pyrethrins to synthetic pyrethroids, their applications have expanded from household insecticides for indoor use against sanitary pests to outdoor use for agriculture, forestry, animal health, termite control, and many other pest situations. However, high fish toxicity and development of pyrethroid resistance in some pests have been cited as common shortcomings of pyrethroids. To overcome these pyrethroid problems such as high fish toxicity, Katsuda and fellow scientists invented silafluofen by introducing a silicone atom into the pyrethroidal chemical structure in 1984. In addition to the high insecticidal activity and low mammalian toxicity, this compound features low fish toxicity, chemical stability under sunlight, in the soil and under alkaline environments. These features make silafluofen unique among pyrethroids. In Japan, silafluofen has been used as an agricultural insecticide for 15 years since 1995 for various plants, especially useful for paddy rice protection because of its low fish toxicity. Over the last 20 years, silafluofen-based termiticides including emulsifiable concentrate (EC) and oil formulations have been widely used in Japan for soil treatment and timber treatments. Additional silafluofen product lines include anti-termitic plastic sheets which are laid under buildings. In this paper, literature on the development of silafluofen and its use in Japan are reviewed. On the other hand, in Thailand, we proceeded with development works of silafluofen-based termiticides from 2005 by starting laboratory efficacy tests and field efficacy tests in Phuket. Both laboratory and field tests showed good efficacy as a soil termiticide, suggesting that the material will perform well for commercial use in high biological hazard environments such as Thailand and can be used in environments close to water where fish toxicity might be a concern with other pyrethroids.

## 1. Introduction: Development of Silafluofen

Natural pyrethrins derived from pyrethrum contain six insecticidal ingredients. Each ingredient is endowed with high selective toxicity, due in part to its excellent insecticidal potency against insects in a small amount and high level of safety to mammals. Because of their potency and safety, pyrethrins [1] have been used for more than 100 years over the world as household insecticides. However, due to their instability to heat, light, and oxygen, there were restrictions in their use in outdoor environments. Since the chemical structures of six insecticidal ingredients of pyrethrins were elucidated in 1958, various structural modifications have been carried out by many countries, leading to the discovery and invention of a variety of synthetic pyrethroids. Initially the pyrethroids such as allethrin [2], phthalthrin [3], furamethrin [4], and phenothrin [5] were obtained mainly by modifications of the alcohol moiety group. These molecular modifications still retained the characteristics of pyrethrins and have been used as household insecticides. Subsequent modifications on the acid moiety created a number of photostable synthetic pyrethroids with improved residual activity that include permethrin [6], deltamethrin [6], fenvalerate [7], fluvalinate [8,9] and bifenthrin [10]. As a result, synthetic pyrethroids are now used in outdoor situations that include agriculture, forestry, animal health, and termite control. Unfortunately, there have been some problems when using pyrethroid compounds as agrochemicals and termiticides due in part to high fish toxicity and chemical instability in the alkaline soils. In addition, there are reports on the emergence of pyrethroid resistance to some pests [11,12].

**Figure 1:**
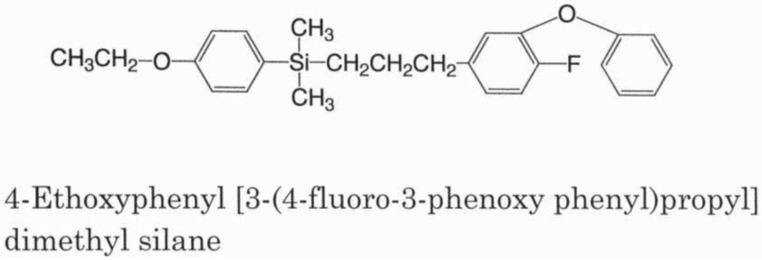
Chemical structure of silafluofen.

To overcome these environmental problems with pyrethroids, additional structural modifications were made that led to the invention of silafluofen (Figure 1) in 1984 [13-16]. The unique feature of silafluofen includes the introduction of a silicone atom into the pyrethroidal chemical structure. This new and novel compound is quite different in structure from the prototype pyrethrins. It is certain, however, that the idea of sila-substitution in pyrethroids emerged in the course of pyrethroid development. Interestingly, the findings on silafluofen were independently published almost at the same time in Japan (1984 in terms of patent application), Germany (1985) [17] and USA (1986) [18]. The mode of action for silafluofen involves its actions on the neuroaxonal sodium channels: it is still considered to be a pyrethroid [19]. While pyrethroids are considered a contact poison, silafluofen is noticeably different and acts both as contact and stomach poisons. In Japan, fish toxicity is classified into the following three classes based on the LC_50_ values for carp: A rank (>10 ppm), B rank (0.5–10 ppm), and C rank (<0.5 ppm). Generally, pyrethroids including bifenthrin are considered highly toxic to fish and belong to C rank except for etofenprox and cycloprothrin that both belong to B rank group. In contrast, silafluofen is the only synthetic pyrethroid in A rank, because of its low toxicity to fish. The unique safety of silafluofen is considered a favorable characteristic and makes it highly valued in its use. Unlike other pyrethroids, such as bifenthrin, which are ester compounds and decompose easily under alkaline conditions, silafluofen is chemically stable. The linkage between carbon and silicon atoms stabilizes silafluofen when applied to alkaline soils. For example, a stability test of test compounds with mortar (pH of water-extract: 12.8) revealed that silafluofen gave a remarkably high recovery rate of more than 90% at 50 °C after 4 week storage whereas bifenthrin almost decomposed within one week [16]. In summary, silafluofen superiority over pyrethroids includes its patent insecticidal activity, low mammalian toxicity described below [20], low fish toxicity [20], mode of action that includes both contact and stomach poisons, and chemical stability in the alkaline soil. The latter three characteristics are very different from those of conventional pyrethroids.

Typical toxicological data of silafluofen:

1. Acute oral toxicity for rat (LD_50_): >5,000 mg/kg (♂,♀);
2. Acute dermal toxicity for rat (LD_50_): >5,000 mg/kg (♂,♀);
3. Acute inhalation toxicity for rat (LC_50_): >6,610 mg/m^3^ (♂,♀);
4. Eye irritation for rabbit: minimally irritating;
5. Skin irritation for rabbit: non irritant;
6. Sensitization for guinea pig: negative;
7. Fish toxicity for carp (LC_50_ after 48 hours): >100 ppm.

## 2. Practical Uses of Silafluofen-Based Termiticides in Japan

**Figure 2:**
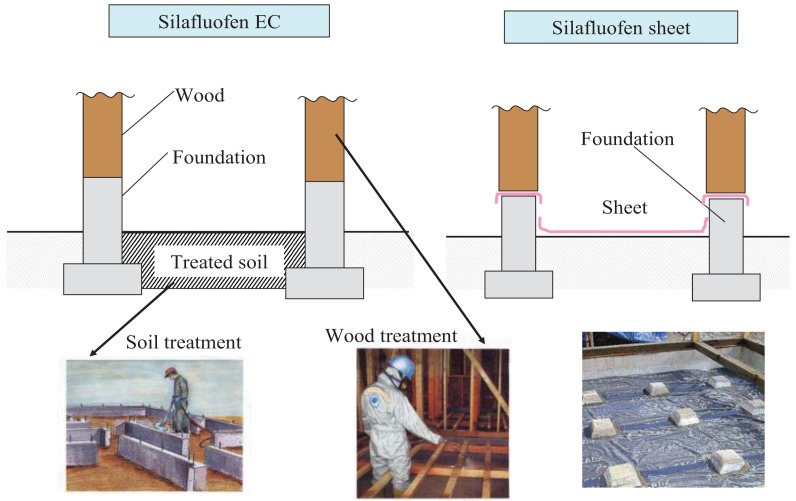
Treatment scenes.

Dainihon Jochugiku and Hoechst in 1988 jointly started developing silafluofen for agricultural use in Japan. After silafluofen received registration as an agrochemical pesticide in 1995, its first agricultural uses included rice paddies, fruit trees, tea trees, and turf Its registration was especially useful in rice paddies because of its low toxicity to fish. Independently during the same period, Dainihon Jochugiku proceeded with the development of silafluofen's non-agricultural uses including termiticides. In Japan, the marketing of silafluofen-based termiticides (Table 1) started in 1991 when emulsifiable concentrates (EC) and oil formulations were applied for soil treatment and timber treatment respectively. Presently EC formulations for timber treatment are widely used. A more recent use (1998) and product line for silafluofen includes its use as anti-termitic plastic sheets impregnated with silafluofen and plastic resin. These sheets are installed under newly-built homes to prevent ingress of termites from the ground. Typical treatment scenes are illustrated in Figure 2. After 20 years, silafluofen continues to be a widely used and successful termiticide product with an excellent reputation in providing effective termite control.

## 3. Efficacy Tests of Silafluofen-Based Termiticides in Thailand

### 3.1. Silafluofen EC Formulation

In accordance with the above EC formulation, this product contains silafluofen 15.0% (w/w) as termiticidal ingredient. The EC is diluted with water, and the dilution is sprayed on the soil or timber at concentrations of 0.10% and 0.15% as silafluofen. Application rates are 5 L/m^2^ for the soil treatment and 0.3 L/m^2^ on the timber treatment, respectively.

In 2008, the silafluofen EC formulation was applied for the Food and Drug Administration (FDA) registration in Thailand, and it was approved in February of 2010.

### 3.2. Laboratory Efficacy Tests

#### 3.2.1. Test Methods

**Figure 3:**
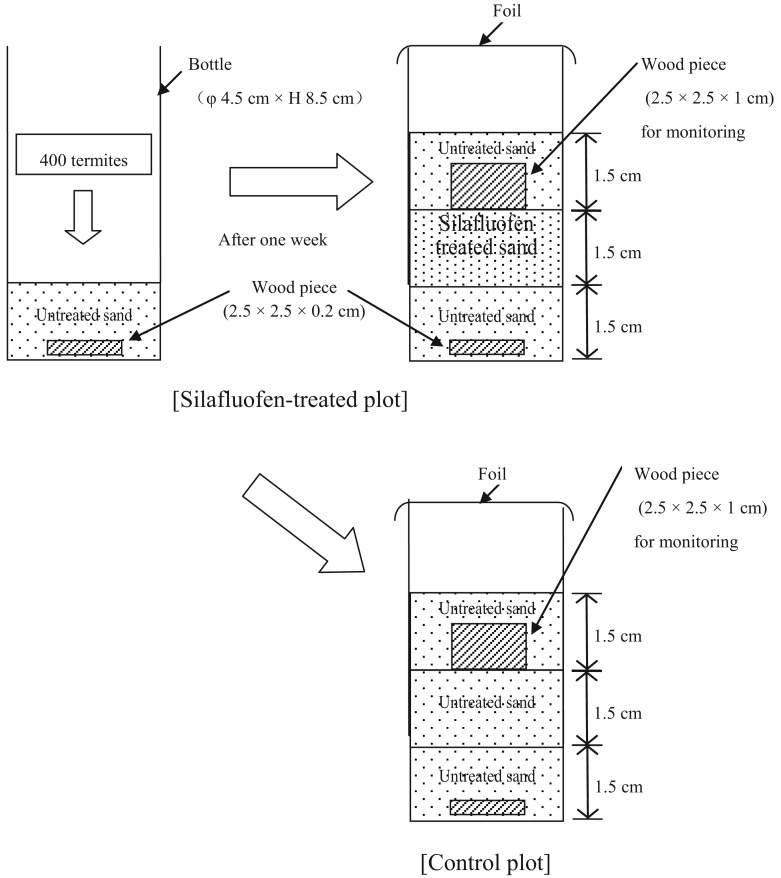
Standard laboratory efficacy test method in Thailand.

Standard Laboratory Efficacy Test in Thailand (see Figure 3) was performed as described previously [21].

#### 3.2.2. Test Results

Test results are shown in Table 2.

Mortality of termites reached 100% within 2 weeks with no or slightly wood weight loss of wood piece at silafluofen plots treated with concentrations of 0.10% and 0.15% as active ingredient.

### 3.3. Field Efficacy Tests

#### 3.3.1. Test Methods

**Figure 4:**
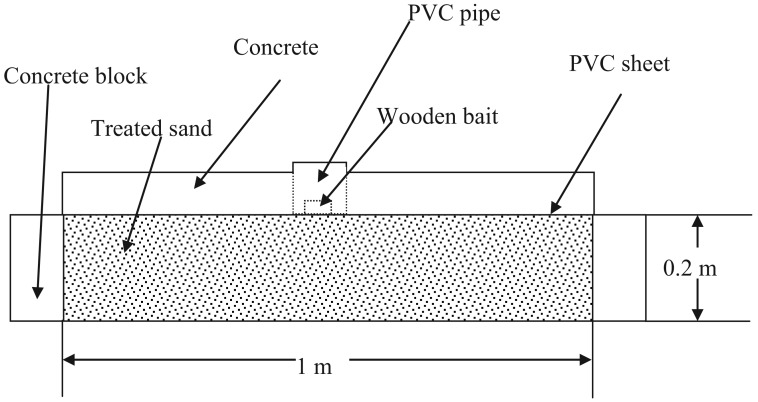
Modified ground board test method in Thailand.

From November of 2005, field efficacy tests were conducted in four replicates in Phuket Province of Thailand according to Standard Method of Royal Forest Department (Modified Ground Board Test) [22]. This includes the following test procedures as shown in Figure 4:

1. Install a test plot (1 × 1 × 0.2 m) with concrete blocks;
2. Fill the installed concrete plot with river sand. Compact by using construction tools;
3. Dilute the test termiticide to required concentrations;
4. Evenly spray the dilution on the surface of the soil, 5 L per 1 plot;
5. Put PVC sheet on the surface of the treated soil;
6. Pour concrete (8 cm thick) on PVC sheet leaving only a hole (Φ: 10 cm) around the PVC pipe (Φ: 10 cm; H: 10 cm) in the middle of the ditch;
7. Cut out the PVC sheet inside the PVC pipe;
8. Put one wooden bait (5 × 5 × 2.5 cm) inside the PVC pipe;
9. Cover the pipe.

#### 3.3.2. Test Results

To evaluate test termiticides, wooden baits on the treated soil were observed for termite damage in comparison with those on the untreated (Control) soil every year after the test installation. Recent test results are shown in Table 3.

Silafluofen at concentrations of 0.10% and 0.15% performed well over 5 years when used under the condition of the Modified Ground Board Test to prevent the underground tunneling of subterranean termites. The large SD value in the control plot after 3 years is considered to be caused by the field test conditions at that time. Nevertheless it is obvious that the field tests in Phuket have confirmed silafluofen's effectiveness in controlling termites for a long time.

## 4. Conclusions

Due to favorable characteristics such as low fish toxicity and chemical stability in the soil and under alkaline environments, silafluofen has been one of the most effective termiticidal ingredients in use in Japan over the last 20 years. In addition to EC and oil formulations for soil treatment and timber treatments, silafluofen product lines include anti-termitic plastic sheets which are laid under buildings. In Thailand, both laboratory and field tests with silafluofen-based EC formulations showed good efficacy as a soil termiticide, suggesting that the material will perform well in commercial use in high biological hazard environments such as Thailand and can be used in environments close to water where fish toxicity might be a concern with other pyrethroids.

## Figures and Tables

**Table 1:** Silafluofen-based termiticides in Japan.

**Kind of Treatment**	**Soil Treatment**	**Timber Treatment**	**Anti-Termitic Sheet**
Termiticidal ingredient	Silafluofen 15.0% (w/w)	Silafluofen 3.0% (w/w) with fungicidal ingredient	Silafluofen ≥0.05% (w/w)
Use method Application rate	Dilute the EC 1 (1 kg) with water (99 L) and spray on the soil (final conc.: 0.15%) 3 L/m^2^ (soil)	Dilute the EC 1 (1 kg) with water (19 L) and spray or paint on the timber (final conc.: 0.15%) 0.3 L/m^2^ (timber)	Lay the sheet on the soil
Start of marketing	From 1991	From 1992	From 1998

1 EC: emulsifiable concentrate.

**Table 2:** Test results of laboratory efficacy tests in Thailand.

**Test Termiticide**	**Mortality of Termites Days after Treatment (%)**							
	1 d	3 d	5 d	7 d	10 d	14 d	30 d	60 d
Silafluofen 0.10%	0	5	10	50	60	100	100	100
Silafluofen 0.15%	5	20	40	100	100	100	100	100
Control	0	0	0	10	10	20	50	80

**Table 3:** Test results of field efficacy tests in Thailand

**Test Termiticide**	**After 3 Years**					**After 5 Years**				
	**Termite Damage(%)**				**Mean ±** **SD (%)**	**Termite Damage (%)**				**Mean ±** **SD (%)**
	1	2	3	4		1	2	3	4	
Silafluofen 0.10%	0	0	0	0	0 ± 0	5	0	0	0	1.3 ± 2.5
Silafluofen 0.15%	0	5	5	0	2.5 ± 2.9	0	0	0	0	0 ± 0
Control	100	10	10	100	55 ± 52	100	80	90	100	92.5 ± 9.6
